# The bidirectional role of neutrophils in the regulation of bone metabolism: a review of research progress and mechanism

**DOI:** 10.3389/fimmu.2026.1868073

**Published:** 2026-10-01

**Authors:** Hengchang Su, Sinian Wang, Weibo Liu, Hao Zhou, Yiying Zhang, Renjun Peng, Zhongmin Chen, Huijie Yu, Zecheng Zhu, Fengsheng Li, Wenhua Liu, Suhe Dong

**Affiliations:** 1 School of Clinical Medicine, Shandong Second Medical University, Weifang, Shandong, China; 2 People's Liberation Army Rocket Force Characteristic Medical Center, Beijing, China; 3 Department of Orthopaedics, the Fourth Medical Centre, Chinese People's Liberation Army (PLA) General Hospital, Beijing, China

**Keywords:** bone metabolism, neutrophil extracellular traps, neutrophils, osteoclastogenesis, osteogenesis, osteoimmunology

## Abstract

Bone homeostasis is maintained through a dynamic balance between osteoblast-mediated bone formation and osteoclast-mediated bone resorption. With the rapid development of osteoimmunology, increasing attention has been directed toward the role of immune cells in the regulation of bone metabolism. Neutrophils, as the most abundant innate immune cells in the bone marrow, are now recognized not only as key effectors of inflammation but also as important regulators of bone microenvironment homeostasis and bone remodeling. This review summarizes current advances in the understanding of neutrophil-mediated regulation of bone metabolism, focusing on their dual roles in osteogenesis and osteoclastogenesis. During the early stages of bone repair, neutrophils may facilitate bone regeneration by clearing necrotic debris, promoting angiogenesis, and modulating mesenchymal stem cells (MSCs) function. However, in pathological settings such as persistent inflammation, metabolic dysfunction, and aging, neutrophil dysregulation can suppress osteogenesis and enhance osteoclastogenesis through the release of inflammatory mediators, reactive oxygen species, and neutrophil extracellular traps (NETs), ultimately contributing to bone metabolic imbalance. Notably, NETs exhibit context-dependent effects in bone remodeling and may either support repair or aggravate bone destruction under different pathological conditions. Overall, neutrophils function as critical intermediates linking immune responses with bone remodeling. Their diverse effects on bone metabolism are largely determined by their activation status and the surrounding microenvironment. Elucidating the mechanisms underlying neutrophil-mediated regulation of bone metabolism may offer new insights and potential therapeutic targets for osteoporosis, inflammatory bone destruction, and impaired bone healing.

## 1 Introduction

Bone metabolism is a highly dynamic process governed by the coordinated balance between osteoblast-mediated bone formation and osteoclast-mediated bone resorption. Maintaining this balance is essential for preserving bone mass, structural integrity, and normal skeletal function. Under physiological conditions, this tightly regulated system allows bone tissue to adapt continuously to changes in mechanical loading and metabolic demands (1). In contrast, disruption of this homeostatic balance may result in a variety of bone-related disorders, including osteoporosis and fracture healing impairment (2, 3).

For a long time, the regulation of bone metabolism was primarily attributed to the coordinated actions of bone-related cells, including osteoblasts, osteoclasts, osteocytes, and bone marrow mesenchymal stem cells, whereas the involvement of immune cells was often regarded as a secondary phenomenon associated with inflammation or tissue injury (4). With the continued development of osteoimmunology, the close interplay between the immune system and bone metabolism has received increasing attention, and immune cells are now recognized as integral regulators of the bone microenvironment (5, 6). Among these, neutrophils are one of the most abundant immune cell populations in the bone marrow and can be rapidly recruited to sites of inflammation or injury, where they participate in local immune regulation through phagocytosis, degranulation, and the release of neutrophil extracellular traps (NETs) (7–9). In addition, neutrophils secrete a variety of cytokines and matrix-related molecules, including TNF-α, IL-1β, IL-6, IL-8, and MMP9, thereby influencing the microenvironment of surrounding tissues (10). Accumulating evidence indicates that both the distribution and functional characteristics of neutrophils are altered to varying degrees under a range of physiological and pathological conditions, such as fracture healing, chronic inflammation, tumor-associated bone metastasis, and aging, suggesting that they may play an important role in maintaining bone metabolic homeostasis (7, 11, 12).

It is worth noting that the role of neutrophils in osteogenesis and osteoclastogenesis is not unidirectional; rather, their functions are highly dependent on the local microenvironment and the underlying disease context (5, 6). During the early stage of bone repair, neutrophils can contribute to the establishment of a pro-regenerative microenvironment by clearing necrotic tissue, promoting angiogenesis, and modulating mesenchymal stem cell behavior (7, 11). In contrast, under conditions such as persistent inflammation, aging, or other pathological states, aberrant neutrophil activation may exacerbate bone resorption while suppressing osteogenesis, thereby disrupting bone homeostasis (5, 6, 9, 13). Therefore, a systematic understanding of the modes of action and regulatory mechanisms of neutrophils in bone metabolism will not only deepen our understanding of the complexity of the osteoimmune microenvironment, but also provide a theoretical basis for the development of new therapeutic strategies for osteoporosis, fracture-healing disorders, and other related bone diseases. Accordingly, this review focuses on the biological characteristics of neutrophils and their roles in the dynamic regulation of osteogenesis and osteoclastogenesis, with the aim of summarizing current advances in our understanding of neutrophil-mediated regulation of bone metabolism.

## 2 Bone metabolism and its immune microenvironment basis

Bone tissue is a highly dynamic metabolic organ that is continuously renewed throughout life. Bone remodeling, the core process of bone metabolism, is characterized by the tightly coordinated coupling of osteoclast-mediated bone resorption and osteoblast-mediated bone formation at the same anatomical site, thereby preserving bone mass and microarchitectural integrity (14–16). Under physiological conditions, these two processes are maintained in relative equilibrium. However, disruption of this coupling, as occurs in estrogen deficiency, chronic inflammation, and aging-associated pro-inflammatory states, can shift the balance toward excessive bone resorption over bone formation, resulting in bone loss, microstructural damage, and ultimately the development of osteoporosis, fragility fractures, and related metabolic bone diseases (15, 17–19).

During bone remodeling, multiple cell types act in concert, among which osteoblasts and osteoclasts constitute the fundamental functional unit of this process (14, 15). Osteoblasts are derived from mesenchymal stem cells and undergo differentiation and maturation under the regulation of signaling pathways such as Runx2, Osterix, and Wnt/β-catenin. Mature osteoblasts secrete type I collagen and a variety of bone matrix proteins, thereby promoting matrix mineralization (16, 20, 21). In contrast, osteoclasts originate from the monocyte-macrophage lineage and differentiate in response to M-CSF and RANKL-RANK signaling. These cells resorb bone by secreting acids and proteolytic enzymes that degrade the mineralized matrix (18, 22). Bone remodeling is a cyclical process with well-defined temporal characteristics and is generally divided into the activation, resorption, reversal, formation, and termination phases (14, 15). During the activation phase, bone-lining cells retract to expose the bone surface, while osteoclast precursor cells are recruited and their differentiation program is initiated (14). This is followed by the resorption phase, during which mature osteoclasts attach to the bone surface, dissolve hydroxyapatite through acidification, and release various proteases to degrade organic matrix components such as collagen. This phase is also associated with the generation of bone resorption markers, including CTX, NTX, and TRACP5b (18, 22, 23). Once resorption is completed, the process enters the reversal phase, which is largely mediated by mononuclear cells that clean the resorbed bone surface and recruit osteoblast precursor cells to the resorption lacuna, thereby preparing the site for subsequent bone formation (15). During the formation phase, osteoblasts synthesize and secrete bone matrix components and promote their mineralization, while releasing bone formation-related markers such as PINP and bone-specific alkaline phosphatase (bone ALP) (16, 20, 23). Finally, in the termination phase, some osteoblasts become embedded within the newly formed bone matrix and differentiate into osteocytes, whereas others remain on the bone surface as lining cells. The bone surface then returns to a relatively quiescent state until the next remodeling cycle begins (14–16).

Following bone injury, local bone homeostasis is rapidly disrupted, and the repair response unfolds through a highly coordinated spatiotemporal program that tightly couples inflammation with regeneration (24, 25). The early phase is characterized by acute inflammation, marked by increased vascular permeability and rapid recruitment of immune cells to the injury site, which together initiate and sustain the subsequent repair cascade (24, 26). Fracture healing generally proceeds through a series of overlapping stages, including hematoma formation and coagulation, inflammatory cell recruitment, vascular ingrowth and soft callus formation, hard callus mineralization, and eventual remodeling into lamellar bone (25, 27, 28). Throughout this process, inflammatory cells not only clear necrotic tissue and potential pathogens, stabilize the hematoma matrix, and remodel the extracellular matrix, but also release pro-inflammatory cytokines and chemokines, including IL-1, IL-6, and TNF-α, to recruit additional immune cells, promote angiogenesis, and establish the inflammatory milieu required for mesenchymal stem cell homing and osteogenic differentiation (24, 26, 29). Nevertheless, although the inflammatory phase is essential for initiating bone repair, excessive or unresolved inflammation can impair osteogenesis and enhance osteoclast activity, thereby delaying bone regeneration and even contributing to structural bone loss (26, 29–31).

The bone marrow microenvironment is not only the primary site of hematopoiesis and immune cell generation, but also a critical platform for bone remodeling (5, 6, 32). The roles of T cells, B cells, and macrophages in regulating RANKL expression, inflammatory mediator production, and osteogenic differentiation have been well established (5, 6, 18, 33). In contrast, the contribution of neutrophils, a major component of the innate immune system, to bone injury, bone regeneration, and bone pathology has only recently begun to receive increasing attention (7, 11, 29). As key effector cells in the early inflammatory response, neutrophils warrant more systematic investigation with regard to their distribution patterns, functional heterogeneity, and regulatory mechanisms within the bone microenvironment (7, 13, 34).

## 3 Biological characteristics of neutrophils and their dynamic changes in the bone microenvironment

### 3.1 Source and differentiation process of neutrophils

Neutrophils are the most abundant innate immune cells in mammals and are primarily derived from hematopoietic stem and progenitor cells in the bone marrow (35, 36). Under steady-state conditions, neutrophil development proceeds through the classical granulopoietic pathway, including granulocyte-monocyte progenitors (GMPs), myeloblasts, promyelocytes, myelocytes, metamyelocytes, and band cells (36, 37). Ultimately, mature neutrophils with multilobed nuclei are generated and released into the peripheral circulation (35, 37).

Under pathological stress conditions, including infection, inflammation, tumors, and radiation injury, the bone marrow can activate emergency granulopoiesis, a process characterized by accelerated expansion of granulocytic precursors and the premature release of immature neutrophils into the peripheral blood (36–38). As a result, the circulating neutrophil pool becomes more heterogeneous in both maturation state and functional phenotype, making emergency granulopoiesis an important source of neutrophil heterogeneity (34, 36, 37, 39). Importantly, although neutrophils at different developmental stages display distinct morphological features, molecular signatures, and effector capacities, current evidence suggests that these differences are more likely to reflect stage-dependent phenotypic plasticity rather than the existence of stable, lineage-committed functional subsets (34, 39, 40).

### 3.2 Formation basis of neutrophil phenotypic heterogeneity

Neutrophils have long been regarded as short-lived, terminally differentiated cells with relatively limited functional diversity (35, 36). However, accumulating evidence has shown that neutrophils display considerable phenotypic heterogeneity under both homeostatic and pathological conditions (34, 39, 40). This heterogeneity does not appear to arise from clearly defined differentiated subpopulations; rather, it is shaped by a combination of intrinsic and extrinsic factors, including maturation stage, cellular age, tissue localization, and microenvironmental cues (34, 39–41).

In the peripheral circulation, mature neutrophils are not a static population but instead undergo dynamic phenotypic changes over time (35, 42, 43). As their residence time in the bloodstream increases, they progressively acquire an “aged” phenotype, typically characterized by reduced CD62L expression, increased CXCR4 expression, nuclear hypersegmentation, and enhanced adhesive and pro-inflammatory potential (40, 42, 44). Under homeostatic conditions, this aging-associated program is tightly regulated by circadian cues and endogenous signaling pathways, and it contributes to the control of neutrophil tissue homing, clearance, and effector function (40, 43, 45).

Moreover, once neutrophils migrate into peripheral tissues, they can acquire distinct tissue-specific phenotypes shaped by local microenvironmental cues (34, 40, 41). Neutrophils in organs such as the lung, spleen, and liver differ from their circulating counterparts in surface marker expression and functional properties, underscoring the critical role of tissue context in determining neutrophil behavior (39–41, 46).

### 3.3 Functional phenotype and characteristics of neutrophils

At the functional level, neutrophils can adopt distinct effector states according to the dominant mode of activation, although these states are more appropriately viewed as dynamic, context-dependent functional programs rather than fixed cell subsets (34, 40, 41). A pro-inflammatory neutrophil state, sometimes described as “N1-like,” is primarily associated with pathogen clearance and amplification of inflammatory responses (7, 40). It is typically characterized by enhanced phagocytosis, increased production of reactive oxygen species (ROS), degranulation, and neutrophil extracellular trap (NET) formation (7, 9, 36). This state is especially prominent during the early stages of acute infection or tissue injury, where it plays a key role in host defense and inflammatory initiation, but may also contribute to bystander tissue damage if excessive or prolonged (7, 36).In contrast, an anti-inflammatory or pro-resolving neutrophil state, often referred to as “N2-like,” is associated with suppression of excessive inflammation, promotion of inflammatory resolution, and support of tissue repair (11, 34, 40, 41). This functional program is generally accompanied by increased expression of anti-inflammatory and pro-repair mediators, including IL-10, TGF-β, arginase-1, specialized pro-resolving mediators (SPMs), and annexin A1, together with reduced release of pro-inflammatory cytokines and attenuated ROS- and NET-mediated cytotoxic effects (11, 41, 47). Within tissue microenvironments, neutrophils in this state may also exhibit enhanced clearance activity and increased production of angiogenic and tissue-reparative factors (11, 41, 47).

Neutrophils exhibit substantial functional plasticity and heterogeneity within different tissue microenvironments.In the tumor microenvironment, for example, tumor-associated neutrophils have been broadly characterized as “N1-like” or “N2-like” phenotypes, representing pro-inflammatory/anti-tumor and immunosuppressive/pro-tumor functional states, respectively (12, 34, 40, 48). However, this dichotomy is increasingly recognized as a simplified functional framework rather than a strict classification of stable neutrophil subsets (12, 34, 40, 48). Instead, neutrophil phenotypes and functions are highly context-dependent and can be shaped by local inflammatory signals and tissue-specific cues. This functional plasticity is not restricted to the tumor microenvironment.During aging, for example, neutrophils may also enter aging-related or functional “disarming” states, characterized by reduced MPO content, altered NET-forming capacity, and remodeling of effector functions (45, 49). Importantly, these changes do not represent a complete loss of function, but rather a shift in the way neutrophils regulate inflammation and maintain tissue homeostasis. Consistent with this concept, single-cell sequencing and other high-resolution profiling approaches have revealed that neutrophils comprise a continuum of dynamic and reversible functional states rather than discrete N1/N2 subsets (12, 34, 39, 41). These states are shaped by developmental stage, tissue localization, and microenvironmental stimuli (12, 34, 39, 41). This state-based perspective is particularly relevant to the bone microenvironment, where distinct neutrophil states may exert diverse and sometimes opposing effects on osteoblasts, osteoclasts, and bone remodeling.

### 3.4 Presence and dynamic changes of neutrophils in the bone microenvironment

Bone marrow serves not only as the primary site for neutrophil production and retention, but also as a central microenvironment in which osteoblasts and osteoclasts coordinately govern bone remodeling (5, 6, 32). In this highly dynamic system, neutrophils are not simply passive immune effector cells; instead, owing to their abundance and marked functional plasticity, they actively contribute to both bone marrow homeostasis and pathological remodeling (34, 36, 40, 49).

Under physiological conditions, bone marrow neutrophils exist across multiple stages of maturation, thereby forming a continuous developmental and functional continuum (36, 37, 46). After completing maturation, most neutrophils are released into the peripheral circulation following a relatively short residence time in the bone marrow, whereas a subset may remain within or return to the bone marrow, where they undergo retention, clearance, or further functional remodeling (37, 42, 44). This dynamic trafficking process is tightly regulated by chemokine signaling, particularly the CXCR4–CXCL12 axis, which plays a central role in neutrophil retention and bone marrow homing under homeostatic conditions (42, 44, 50).

Beyond their numerical predominance, bone marrow neutrophils under homeostatic conditions may exert a tonic restraining effect on bone remodeling (5, 6, 34, 49). Emerging evidence suggests that quiescent or non-activated neutrophils can negatively regulate osteogenic activity through direct interactions with osteoblast-lineage cells and their progenitors (51). In particular, co-culture studies using bone marrow neutrophils with BMSCs or MC3T3-E1 cells have indicated that the inhibitory effect on osteogenic differentiation is dependent on direct cell–cell contact, as physical separation of the two cell populations markedly attenuates this effect, arguing against a mechanism mediated exclusively by soluble factors (51) (**Figure 1**). This contact dependence raises the possibility that membrane-associated or juxtacrine ligand–receptor interactions contribute to neutrophil-mediated regulation of osteogenesis (51). However, the specific molecular mediators remain poorly characterized. Cell–cell communication analyses have suggested candidate interactions, including HGF–MET and OSM-related signaling, which may participate in neutrophil–MSC communication, but these predictions have not yet been established as causal mechanisms in this context (52). Direct validation using ligand/receptor blockade, genetic perturbation, or recombinant protein-based gain- or loss-of-function approaches will therefore be required to determine whether these candidate pathways mediate the contact-dependent suppression of osteogenic differentiation (52).

**Figure 1:**
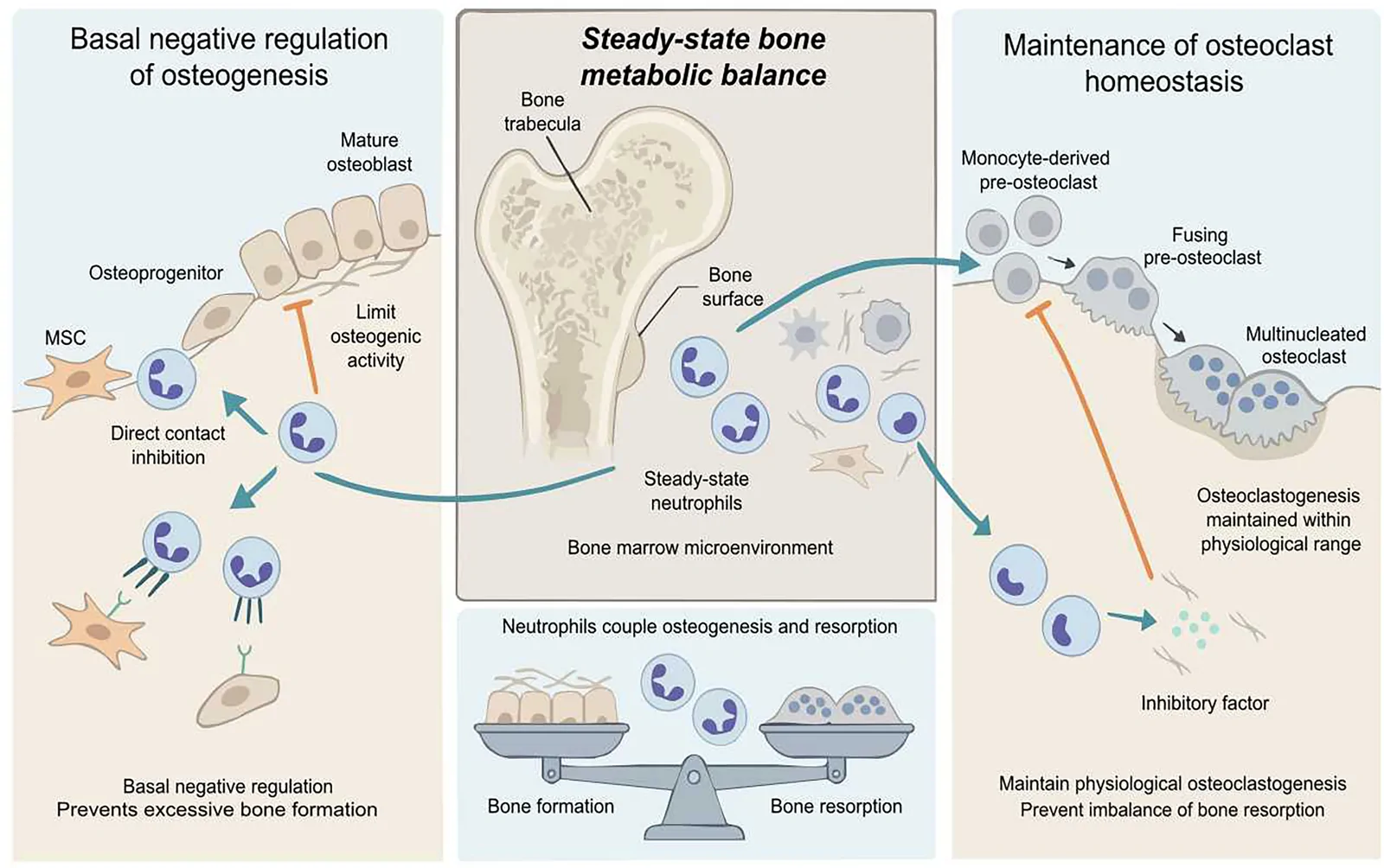
The role of neutrophils in maintaining bone metabolic homeostasis under steady-state conditions. Under steady-state conditions, neutrophils residing in the bone marrow microenvironment contribute to the maintenance of bone metabolic balance by coordinating osteogenesis and osteoclastogenesis within a physiological range. On the osteogenic side, steady-state neutrophils may exert a basal restraining effect on mesenchymal stem cells (MSCs), osteoprogenitors, and osteoblast-lineage cells, limiting excessive osteogenic activity through contact-dependent or microenvironment-mediated regulation. On the osteoclastogenic side, neutrophils indirectly contribute to osteoclast homeostasis by helping preserve the homeostatic marrow milieu and maintaining osteoclast formation within a physiological range, preventing excessive bone resorption. Overall, neutrophils are depicted as homeostatic regulators that participate in the coupling of bone formation and bone resorption in the steady-state bone marrow niche. MSCs, mesenchymal stem cells.

At the same time, neutrophil-related populations at particular activation or differentiation states may also help limit osteoclast precursor expansion and osteoclastogenesis through inhibitory signals, thereby contributing to the prevention of excessive bone resorption (5, 6, 49). Although these mechanisms remain incompletely understood and the available evidence is still limited, such findings support the view that neutrophils are not merely inflammatory reserve cells in the bone marrow microenvironment, but may instead participate in maintaining the dynamic equilibrium between osteogenesis and osteoclastogenesis as part of the broader bone remodeling homeostasis network (5, 6, 34, 49).

Collectively, under steady-state conditions, bone marrow neutrophils appear to function as homeostatic regulators that help maintain the balance between osteogenesis and osteoclastogenesis in the bone marrow niche (**Figure 1**). The specific roles of neutrophils in regulating osteogenesis and osteoclastogenesis are discussed in the following sections.

Moreover, the dynamic equilibrium of bone marrow neutrophils is markedly altered under stress conditions (36–38). Emergency granulopoiesis can be rapidly activated, resulting in expansion of the neutrophil pool together with increased heterogeneity in maturation status (34, 36–38). At the same time, inflammatory and stress-related cues within the bone marrow microenvironment can drive functional reprogramming of neutrophils, thereby reshaping their phenotypes and effector functions (34, 36, 46). These changes are essentially adaptive responses that enable neutrophils to rapidly eliminate harmful stimuli and maintain microenvironmental stability (36–38).Nevertheless, this adaptive response is not without limits. When neutrophils are excessively recruited or persistently activated, their initially protective effects may gradually convert into detrimental influences on the bone microenvironment (7, 9, 11, 34). Under such conditions, neutrophils can interfere with local bone homeostasis through inflammatory mediator release, protease-dependent extracellular matrix remodeling, and ROS- and NET-associated tissue injury, thereby exerting important effects on bone remodeling (7, 9, 11, 13) In contrast, during the resolution phase of inflammation and subsequent tissue repair, specific neutrophil functional states may indirectly support bone regeneration, partly through the promotion of vascular reconstruction (11, 26). These mechanisms will be discussed in more detail in the following sections.

At the phenotypic level, bone marrow neutrophils can exhibit state-dependent variation in granule protein stores and effector functions (34, 36, 40). Mature neutrophils are generally rich in myeloperoxidase (MPO) and other granule proteins, and are characterized by strong respiratory burst capacity and pronounced pro-inflammatory activity (36, 53). Under conditions such as radiation injury or sustained stress, however, some neutrophils may show reduced MPO content or activity, impaired ROS-generating capacity, and broader effector remodeling (40, 44, 45). These alterations are more likely to reflect a transition from an acute inflammatory phenotype toward an aged or functionally “disarmed” state than simple cellular exhaustion (43–45).

In addition, the bone microenvironment can reshape neutrophil survival, maturation state, functional phenotype, and migratory behavior through a variety of local signals (32, 34, 40, 42). Emerging evidence suggests that, in pathological settings such as bone metastasis and multiple myeloma, microenvironment-derived cues can drive neutrophils toward immature, immunosuppressive, or tumor-supportive functional states, thereby contributing to disease progression (12, 34, 40, 48). In multiple myeloma, for example, inflammatory stromal cells in the bone marrow may reprogram mature neutrophils to acquire pro-tumor functions and establish reciprocal signaling interactions with stromal components (12, 48).Under physiological conditions, bone marrow neutrophils are primarily retained through the CXCR4–CXCL12 axis, with CXCL12 being produced largely by osteoblast-lineage cells and bone marrow mesenchymal stromal cells (32, 42, 50, 54, 55). This indicates that neutrophil retention and trafficking within the bone marrow are not autonomous processes, but are instead tightly linked to the structural and regulatory organization of the bone microenvironment (32, 42, 50, 54). By contrast, CXCR2 signaling promotes neutrophil egress into the circulation, whereas G-CSF mobilizes bone marrow neutrophils in part by enhancing CXCR2-dependent trafficking and/or antagonizing CXCR4-mediated retention (50, 56, 57). Collectively, this microenvironment-driven regulation of neutrophil survival, maturation, phenotype, and migration likely underlies the distinct effects of bone marrow neutrophils across different pathological stages of bone disease (32, 34, 40, 42).

Overall, bone marrow neutrophils are characterized by dynamic changes in cell number, maturation state, and functional phenotype. This phenotypic remodeling and functional plasticity position neutrophils as an important cellular bridge between immune responses and bone remodeling, while also providing a basis for understanding their roles in osteoclast activation and the regulation of osteogenesis.

## 4 The role of neutrophils in bone formation

### 4.1 Evidence that neutrophils promote osteogenesis

#### 4.1.1 “Repair initiation function” of neutrophils in the inflammatory phase of early fracture

Fracture repair is a highly coordinated dynamic process in which the early inflammatory phase is essential for the proper initiation of subsequent osteogenic responses (25–27). As the first immune cells recruited to the fracture site, neutrophils rapidly accumulate within the injured area within hours after fracture (27–29). They contribute to early repair not only by clearing pathogens and necrotic debris through phagocytosis and pro-inflammatory signaling, but also by supporting clot stabilization and the formation of the initial fracture hematoma, which serves as a provisional scaffold for subsequent cell migration and tissue repair (11, 25–29). In addition, emerging evidence suggests that neutrophils may participate in the assembly of a provisional extracellular matrix enriched in fibronectin and other matrix-associated components during the early phase of injury (11). Together with the fibrin network, this transient matrix may provide structural support for the adhesion and recruitment of immune and repair-related cells, while also facilitating the local retention of growth factors and the establishment of a pro-regenerative microenvironment (11, 26).

During the early inflammatory phase, infiltrating neutrophils are predominantly pro-inflammatory and release cytokines and chemokines, including TNF-α, IL-8, and CCL2, which rapidly amplify local inflammation and promote the recruitment of additional immune cells (7). In doing so, they help convert the injured tissue from a hemorrhagic and necrotic state into a microenvironment permissive for repair (26). As inflammation progresses, neutrophils undergo dynamic phenotypic transitions that help support the subsequent shift from inflammatory initiation to regeneration-dominated repair.

These observations indicate that neutrophils do not merely function as executors of the early inflammatory response after fracture, but also provide essential initiating conditions for subsequent angiogenesis, mesenchymal stem cell recruitment, and immune microenvironment remodeling (7, 11, 26, 29). In this way, they serve as a critical hub linking acute injury to the establishment of a regenerative microenvironment (26). However, persistent or excessive neutrophil activation may disturb inflammatory homeostasis and impair the osteogenic process (29). Therefore, precise temporal regulation of neutrophil activity within a defined window is crucial for the proper initiation of the fracture repair cascade.

#### 4.1.2 Repair/deinflammatory: neutrophils promote osteogenesis by regulating angiogenesis

Beyond the initial inflammatory phase, evidence from intermediate-stage fracture healing further suggests that the gradual transition toward a repair-associated neutrophil state may facilitate inflammatory resolution and vascular–osteogenic coupling (58).Angiogenesis is tightly coupled to osteogenesis and is an essential component of bone repair and remodeling (25). After early inflammatory debridement and initial establishment of the reparative microenvironment, a subset of neutrophils may gradually shift from a strongly pro-inflammatory phenotype toward a repair-associated state and persist within the fracture niche (7, 11). These neutrophils can release pro-angiogenic mediators, including VEGF and matrix metalloproteinases involved in extracellular matrix remodeling (7, 11, 59). VEGF is a key regulator of bone angiogenesis and supports osteogenesis indirectly by promoting vascular ingrowth and ensuring adequate oxygen and nutrient supply to osteogenic cells (59–61). Because osteoblasts and their progenitors also express VEGF receptors, VEGF may additionally participate in the direct regulation of osteogenic differentiation (59–61). Meanwhile, neutrophil-derived MMP-9 can facilitate vascular invasion into the repair tissue by remodeling local extracellular matrix barriers and modulating the tissue availability of VEGF (62, 63). Therefore, the pro-osteogenic contribution of neutrophil-derived MMP-9 is more likely to operate through vascular–osteogenic coupling than through direct stimulation of osteoblast differentiation (62, 63).

Through their effects on angiogenesis and vascular remodeling, neutrophils can indirectly support bone formation by improving the nutritional and signaling milieu required for osteogenesis (25–27, 59). Nevertheless, this pro-reparative effect is strongly contingent on neutrophil functional state, as excessive inflammatory activation or persistent oxidative stress may destabilize the vascular network and impair osteogenesis (26). Thus, dynamic phenotypic transitions of neutrophils appear to be a key determinant of vascular–osteogenic coupling during bone repair.

#### 4.1.3 Neutrophils regulate mesenchymal stem cells behavior to indirectly promote osteogenesis

Osteoblasts are primarily derived from bone marrow mesenchymal stem/stromal cells (BMSCs), and the migration, expansion, and osteogenic differentiation of MSCs are key determinants of bone formation efficiency (20, 64, 65). Given their central role in coordinating inflammation and tissue repair, neutrophils may influence osteogenesis in part by modulating MSCs behavior and MSC-mediated bone regeneration (5, 7, 11, 26).

First, neutrophils may promote the recruitment and homing of MSCs to sites of injury or implanted biomaterials through the release of chemokines, growth factors, and matrix-remodeling mediators (5, 7, 11, 26). As inflammation resolves, a subset of neutrophils gradually shifts from a predominantly pro-inflammatory state toward a pro-resolving or repair-associated phenotype (7, 11, 47). During this process, neutrophil-derived mediators involved in chemotaxis, angiogenesis, and extracellular matrix remodeling, including CXCL12-related signaling, VEGF, and matrix metalloproteinases such as MMP-9, may help remodel the local microenvironment, reduce barriers to cell migration, and enhance the directed recruitment of MSCs to the injured area (5, 7, 47, 59, 62). In an IL-8-induced ectopic endochondral ossification model, repair-associated neutrophils have also been implicated in BMSC recruitment through neutrophil-derived SDF-1α and the SDF-1/CXCR4 axis, thereby facilitating ectopic bone formation (66). In parallel, repair-associated neutrophil responses may also facilitate the transition of macrophages from a pro-inflammatory to an anti-inflammatory phenotype, thereby creating a microenvironment more favorable for MSCs survival and osteogenic differentiation (67, 68). Anti-inflammatory macrophages, in turn, can support MSCs-mediated repair through the secretion of trophic factors and suppression of excessive inflammation (65, 67, 68). Second, neutrophils may support the osteogenic differentiation of MSCs through paracrine mechanisms (5, 7, 11, 26). Neutrophil-derived mediators involved in tissue repair, including angiogenic factors, growth-related signals, and selected chemokines, have been reported to influence osteogenesis-associated pathways in MSCs, such as Wnt/β-catenin, BMP/Smad, and ERK/MAPK signaling (5, 7, 11, 20, 59). As a result, the expression of osteogenic markers, including Runx2, ALP, COL1A1, and OCN, may be enhanced, thereby promoting differentiation of MSCs toward the osteoblastic lineage (5, 7, 20, 59, 65). In addition, neutrophil-mediated remodeling of the immune microenvironment may indirectly enhance MSC-driven bone formation by facilitating angiogenesis–osteogenesis coupling (11, 25, 26, 59). Newly formed vessels not only provide essential oxygen, nutrients, and migratory pathways for MSCs, but also generate a local niche enriched in pro-osteogenic signals (25, 59–61). Through reciprocal interactions with MSCs, this vascular niche can further establish a positive feedback loop that amplifies local osteogenic activity and supports the sustained progression of bone repair (59–61, 65). Consistent with this concept, studies using critical-sized calvarial defects have shown that early neutrophil recruitment contributes to the initiation of repair and subsequent regenerative cell recruitment, as neutrophil depletion is associated with reduced new bone formation (66). Similar pro-regenerative effects have also been reported in tibial implant models, in which early neutrophil recruitment may support BMSC recruitment and early osteogenic responses at the implant interface, with neutrophil depletion impairing peri-implant bone formation and osseointegration (69).

In summary, neutrophils support bone regeneration by modulating MSCs recruitment, survival, and osteogenic differentiation, while simultaneously coordinating angiogenesis and immune microenvironment remodeling (11, 26, 34, 59). This neutrophil–MSCs coupling mechanism offers an important perspective for understanding the transition from late inflammation to the regenerative phase of bone repair (26, 34, 59, 65).

### 4.2 Mechanism of neutrophil inhibition of osteogenesis

Although neutrophils can facilitate osteogenesis during specific stages of bone repair by promoting angiogenesis and remodeling the immune microenvironment, increasing evidence suggests that their effects on osteogenesis are not uniformly protective but instead highly context-dependent (7, 11, 26, 34). Under conditions of excessive or sustained infiltration, or in pathological settings such as metabolically dysregulated microenvironments, neutrophils may shift from a pro-reparative phenotype to a pro-inflammatory and tissue-damaging state, thereby suppressing osteogenesis (5, 7, 9, 26, 34). In light of this, the following sections systematically summarize the major mechanisms through which neutrophils inhibit osteogenesis and discuss their pathophysiological significance.

#### 4.2.1 Neutrophil-derived inflammatory factors directly inhibit osteoblast differentiation and function

In inflammatory or tissue-damage microenvironments, neutrophils are rapidly recruited and activated, leading to the release of large amounts of pro-inflammatory cytokines that can act directly on osteoblasts and their progenitor cells, thereby inhibiting osteogenic differentiation and maturation (5, 7, 13, 26).

Among these mediators, TNF-α represents one of the most important inhibitory signals affecting osteogenesis (5, 26, 70). TNF-α can suppress the transcription of osteogenesis-related genes through activation of the NF-κB and MAPK pathways, while also negatively regulating Wnt/β-catenin signaling, thereby weakening the core transcriptional axis that drives osteogenic differentiation (70–73). In addition, TNF-α has been reported to promote the ubiquitination and degradation of Runx2 by upregulating the E3 ubiquitin ligases Smurf1 and Smurf2, and to inhibit Osterix expression, resulting in a dual blockade of the osteogenic program at both the transcriptional and protein-stability levels (72–75). Functionally, these changes are manifested by reduced ALP activity, impaired mineralized nodule formation, and increased apoptosis of osteoblast-lineage cells (70, 71, 73–75).

IL-1β is an important inflammatory mediator that can inhibit osteogenesis under persistent inflammatory conditions, primarily through activation of the MAPK and NF-κB pathways (5, 24, 26, 73). Sustained IL-1β signaling interferes with the recruitment and osteogenic commitment of precursor cells, suppresses the expression of key osteogenic transcription factors such as Runx2 and Osterix, and thereby disrupts the transcriptional program required for osteoblast differentiation (5, 24, 72, 73). Moreover, prolonged IL-1β exposure may drive osteoblast-lineage cells into a stress-associated inflammatory state, leading to functional impairment and even cell death, and ultimately reducing osteogenic potential at both the cellular and differentiation levels (24, 26, 73).

Under sustained inflammatory conditions, IL-6 can impair osteogenic differentiation and maturation predominantly through JAK/STAT3 signaling, while also interacting with other downstream pathways such as MAPK- and SHP2-related signaling (5, 24, 26). Prolonged STAT3 activation disrupts the signaling balance required for MSC commitment toward the osteoblastic lineage and is associated with reduced expression of mature osteogenic markers, including ALP, COL1A1, and OCN (5, 26). As a consequence, extracellular matrix mineralization and terminal differentiation are suppressed, and the osteogenic program may remain in a relatively immature state (24, 26).

Collectively, TNF-α, IL-1β, and IL-6 are well-established inflammatory mediators that can impair osteoblast function through effects on osteogenic transcription, protein stability, cell fate decisions, and matrix mineralization, involving NF-κB-, MAPK-, and JAK/STAT3-related signaling pathways (5, 24, 26, 70, 73). However, although neutrophils can produce or release these cytokines under inflammatory conditions, much of the mechanistic evidence for their inhibitory effects on osteogenesis is derived from studies of the cytokines themselves or from broader inflammatory models rather than from neutrophil-specific experimental systems. Thus, these findings suggest that neutrophil-derived TNF-α, IL-1β, and IL-6 may contribute to the suppression of osteogenesis within inflammatory microenvironments, but their relative and direct contribution to neutrophil-mediated osteogenic inhibition remains to be fully established.

#### 4.2.2 Inhibition of osteogenesis mediated by neutrophil reactive oxygen species and oxidative stress

In inflammatory or tissue-damage microenvironments, neutrophils are rapidly recruited and activated, triggering a respiratory burst that produces large amounts of reactive oxygen species (ROS), a key component of their host-defense machinery (7, 26, 76). When produced in excess, however, ROS can directly injure local cells and tissue structures and simultaneously disturb osteogenesis-related signaling and microenvironmental homeostasis during bone repair, thereby potentially contributing to sustained suppression of osteogenesis (26, 76, 77).

In chronic inflammatory conditions, neutrophils are persistently stimulated by pathogens and inflammatory mediators, resulting in sustained respiratory burst activity and prolonged exposure of the local microenvironment to elevated high levels of ROS (7, 9, 26, 78). Excessive ROS not only directly damages surrounding tissues, but also promotes NET formation and MPO-dependent oxidative reactions, further amplifying local inflammatory injury (8, 9, 26, 53). As a result, a self-perpetuating ROS–NETs–MPO amplification circuit may emerge, potentially contributing to persistent oxidative stress and impaired tissue repair (8, 9, 53, 79). Meanwhile, chronic oxidative stress can maintain macrophages in a pro-inflammatory state and hinder their transition toward repair-associated phenotypes, thereby delaying inflammatory resolution and further disrupting the bone repair microenvironment (67, 68, 78). Through this broader immune dysregulation, neutrophil-derived oxidative stress may indirectly reinforce the suppression of osteogenesis (26, 67, 68, 78).

From the perspective of osteoblast-lineage cells, excessive ROS can impair impairs osteogenesis through multiple mechanisms (26, 77, 80, 81). Excessive ROS has been reported to suppresses the expression of key osteogenic regulators, including Runx2 and Osterix, reduces ALP activity and OCN production, and impairs collagen synthesis and extracellular matrix deposition, thereby inhibiting osteoblast differentiation and mineralization (26, 80–82). Meanwhile, extracellular ROS can directly damage cell membranes through lipid peroxidation, whereas intracellular ROS overload may induce osteoblast apoptosis via the mitochondria-dependent caspase-9/caspase-3 pathway (26, 82). In addition, oxidative stress promotes nuclear translocation of FoxO transcription factors, which can competitively divert β-catenin away from canonical Wnt signaling toward antioxidant transcriptional programs. This shift may weakens Wnt/β-catenin-dependent osteogenic signaling and further compromises osteoblast differentiation (80, 81, 83, 84).

Upstream of osteoblast dysfunction, neutrophil-derived ROS may also affect the fate of BMSCs (77, 80, 81). Excessive oxidative stress can suppresse osteogenic commitment of BMSCs and may induce apoptosis of BMSCs as well as early osteoblast-lineage cells, thereby potentially reducing the regenerative pool of bone-forming cells (77, 80–82). In settings of stress granulopoiesis, including G-CSF-driven neutrophil expansion, enhanced neutrophil activity has been associated with oxidative disruption of the bone marrow niche together with osteoblast suppression and reduced expression of osteoblast-associated factors (57, 85). Collectively, these observations suggest that neutrophil-associated oxidative stress may contribute to loss of bone-forming cells and impaired osteogenesis, although the extent to which these effects are directly attributable to neutrophil-derived ROS remains to be fully established (57, 77, 80, 81, 85).

These findings provide a rationale for therapeutic strategies targeting the neutrophil ROS–oxidative stress axis (26, 77, 80, 81). In addition to endogenous antioxidant enzymes such as SOD and CAT, a variety of SOD-like and CAT-like enzyme-mimetic materials have recently been developed and shown to attenuate local oxidative stress by scavenging superoxide anion and H_2_O_2_, thereby improving the osteogenic microenvironment (77, 80, 86, 87). Experimental evidence further suggests that oxidative injury associated with neutrophil expansion may be at least partially reversible, as ROS scavenging can reduce apoptosis of osteoblast-lineage cells under stress conditions (26, 57, 80, 85). Moreover, transcriptomic analyses indicate that osteoblasts in osteoporotic bone tissue may display a distinct oxidative stress signature, consistent with persistent ROS burden in the bone microenvironment (77, 80, 81). Given that neutrophils represent an important source of ROS within the bone marrow niche, neutrophil-associated oxidative stress may contribute to broader immune–bone remodeling crosstalk that amplifies osteogenesis impairment. Taken together, current evidence supports a potential role for neutrophil-derived oxidative stress in suppressing osteoblast function and reinforcing pathological inhibition of bone formation through immune–bone coupling, although its specific contribution relative to other cellular sources of ROS remains to be clarified (26, 77, 80, 81).

#### 4.2.3 Effects of cytotoxic inhibition mediated by neutrophil release NETs on osteogenesis

NETs represent an important effector mechanism by which neutrophils regulate bone metabolism. Increasing evidence suggests that excessive NET formation is associated with impaired osteogenesis and may contribute to bone loss and delayed bone repair under inflammatory or metabolic stress conditions (9, 11, 26, 88–90). Specifically, NETs can limit bone formation by directly damaging osteoblasts and osteoprogenitor cells, impairing the osteogenic potential of BMSCs and tissue-resident MSCs, and sustaining a pro-inflammatory bone microenvironment that is unfavorable for repair (11, 26, 88–90). Thus, NET formation represents an important mechanism linking neutrophil activation to the suppression of bone formation and disruption of bone remodeling homeostasis (9, 88–90).

The detailed mechanisms underlying the effects of NETs on osteoblasts, BMSCs, and the bone microenvironment, as well as their roles in different bone diseases, will be discussed separately in Section 6.

#### 4.2.4 Neutrophil-mediated inhibition of osteogenesis through metabolite–enzyme interactions

In addition to classical immune effector mechanisms involving inflammatory mediators, ROS, and NETs, metabolically dysregulated microenvironments may also contribute to neutrophil-associated inhibition of osteogenesis through a noncanonical pathway of metabolite-mediated enzyme inhibition. In a hyperglycemic bone defect model, recent studies in hyperglycemic bone defect models have identified a PAD4–4-GBA–ALP inhibitory axis, in which neutrophil-associated metabolic reprogramming was reported to target key osteogenic enzymes and impair bone formation. This finding offers a novel mechanistic explanation for impaired bone repair under conditions of disordered glucose metabolism (91, 92).

In the high-glucose microenvironment, neutrophils recruited to the bone injury area underwent metabolic and signaling reprogramming, and the ERK/JNK pathway showed sustained activation, thereby promoting increased expression and activity of arginine deaminase PAD4 (91). PAD4-mediated acceleration of arginine metabolism promotes neutrophil synthesis and extracellular release of 4-guanidinobutyric acid (4-GBA) (91). This metabolite was reported to directly interact with ALP on the surface of BMSCs with high affinity, occupying its catalytic pocket and inhibiting pyrophosphate hydrolysis, resulting in insufficient local inorganic phosphorus supply (91).

Due to the pivotal role of ALP activity in the early osteogenic commitment and mineralization initiation stage, the 4-GBA-mediated inhibition of ALP function may subsequently impair the osteogenic transcriptional program, which is manifested as the down-regulation of the expression of key osteogenic transcription factors such as Runx2 and Sp7, the restriction of osteogenesis-related gene network activation, and the significant reduction of mineralized nodule formation, which ultimately leads to a simultaneous decrease in osteogenic differentiation efficiency and mineralization capacity. In animal models, intervention with neutrophil PAD4 or reducing 4-GBA loading can partially restore ALP activity and MSCs osteogenic capacity, while exogenous supplementation with 4-GBA can replicate the osteogenic inhibitory phenotype under high glucose conditions, supporting a causal role for this pathway in the hyperglycemic model examined (91).

Taken together, evidence from hyperglycemic bone defect models suggests that the hyperglycemic microenvironment may promote neutrophil metabolic reprogramming to establish a cross-cellular PAD4–4-GBA–ALP inhibitory axis, which directly compromises the initiation of osteogenesis by limiting mineral substrate availability. This mechanism offers a potential molecular framework for understanding diabetes-related delayed bone healing and impaired bone formation, but whether this pathway operates similarly in other bone disorders or inflammatory and metabolic conditions remains unclear. Accordingly, neutrophil PAD4 and 4-GBA–ALP interactions may represent context-dependent therapeutic targets in metabolically abnormal bone repair, although their broader therapeutic relevance requires further validation (91).

#### 4.2.5 Contact-dependent osteogenesis inhibition of bone marrow neutrophils in non-inflammatory homeostasis

Even in the non-inflammatory homeostatic bone marrow microenvironment, quiescent neutrophils may exert a tonic restraining effect on osteoblast-lineage cells. Unlike the inhibitory mechanisms observed under inflammatory conditions, this effect does not appear to rely primarily on classical pro-inflammatory cytokines or NET formation, but is more likely mediated through direct contact-dependent interactions with osteoblasts and their progenitors (51).

Under homeostatic conditions, bone marrow neutrophils remain in sustained spatial proximity to osteoblast-lineage cells. Available evidence suggests that reduction or clearance of neutrophils from the bone marrow niche may be associated with enhanced bone formation and increased bone mass, whereas their physiological presence may contribute to a tonic restraint on osteogenic activity. This pattern supports the concept that neutrophils may serve as a basal negative regulator during steady-state bone remodeling. Importantly, the inhibitory effect is markedly weakened when direct cell–cell contact is interrupted, indicating that it is at least partly contact-dependent (51).

Although available evidence suggests that quiescent neutrophils in the steady-state bone marrow microenvironment can restrain osteogenesis through direct contact with osteoblast-lineage cells, the underlying molecular basis and spatial regulatory mechanisms remain largely unclear. In contrast to the cytokine-, ROS-, or NET-mediated “injury-type inhibition” observed under inflammatory conditions, this contact-dependent effect is more likely to reflect a physiological rate-limiting mechanism that constrains osteogenesis during normal bone remodeling, thereby preventing excessive local bone formation and maintaining bone metabolic homeostasis.

Under pathological conditions, persistent or dysregulated neutrophil activation may contribute to the disruption of bone metabolic homeostasis through multiple mechanisms affecting both bone formation and resorption. The major mechanisms discussed in this section are summarized in **Figure 2**.

**Figure 2:**
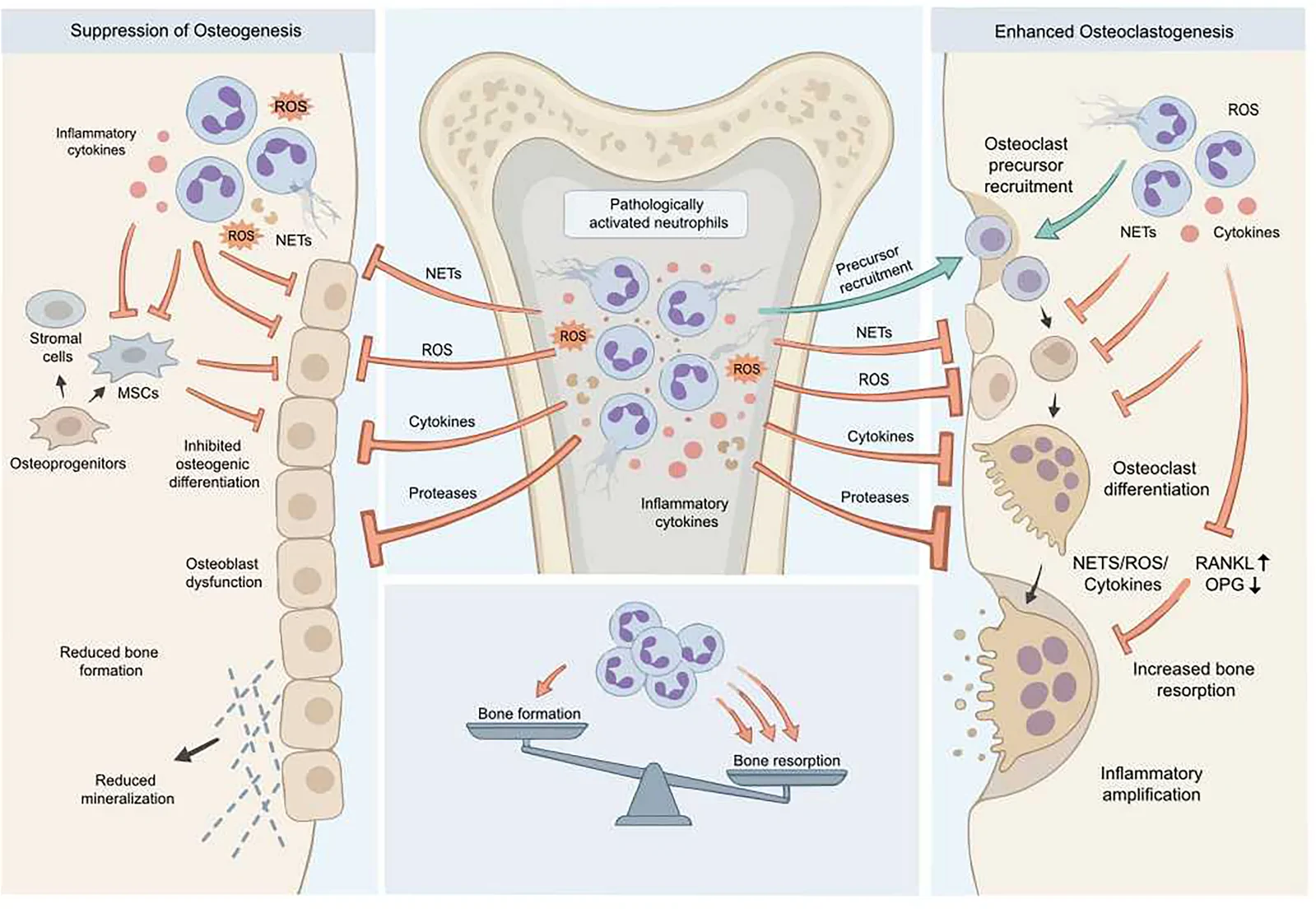
The neutrophils disrupt bone metabolic homeostasis in pathological state. Under pathological conditions, neutrophils become excessively activated within the bone marrow microenvironment and shift toward a pro-inflammatory, tissue-damaging state. The activated neutrophils release inflammatory cytokines, ROS, NETs and proteases, which collectively suppress osteogenic differentiation of MSCs and osteoprogenitors, impair osteoblast function, and reduce bone formation and mineralization. The neutrophil-derived mediators promote osteoclast precursor recruitment and differentiation, increase the RANKL/OPG ratio in the local microenvironment, and enhance osteoclast-mediated bone resorption. Collectively, these processes shift bone remodeling toward impaired bone formation and excessive bone resorption, ultimately leading to pathological bone loss and bone metabolic imbalance. MSCs, mesenchymal stem cells; ROS, reactive oxygen species; NETs, neutrophil extracellular traps; RANKL, receptor activator of nuclear factor-κB ligand; OPG, osteoprotegerin.

## 5 Effect of neutrophils on osteoclasts

The regulatory effects of neutrophils on osteoclasts are most evident under pathological conditions, especially in the settings of inflammation and endocrine imbalance (89). In these contexts, neutrophils can promote osteoclast precursor recruitment, differentiation, and activation through multiple pathways, including the release of pro-inflammatory mediators such as RANKL, IL-1, TNF-α, and IL-6, together with ROS production, NET formation, and secretion of chemokines such as CXCL1 (93–95). Through these mechanisms, neutrophils contribute to increased bone resorption in inflammation-associated bone destruction and estrogen deficiency-related bone loss (94, 96). However, recent studies suggest that neutrophils at different maturation stages or in different functional states within the bone marrow microenvironment may not exert identical effects on osteoclastogenesis (97, 98). This highlights the complexity of neutrophil–osteoclast crosstalk and the need for further mechanistic clarification.

### 5.1 Mechanisms by which neutrophils promote osteoclastogenesis

#### 5.1.1 Neutrophils promote osteoclast differentiation through the RANKL signaling axis

Activated neutrophils can express membrane-bound RANKL (mRANKL) on their surface, which directly binds to RANK on osteoclast precursor cells, thereby promoting their differentiation into mature osteoclasts and enhancing bone resorptive activity (94, 99, 100). Through this mechanism, neutrophils act not only as amplifiers of inflammation within the inflammatory microenvironment, but also as direct regulators of osteoclastogenesis (101).In addition to directly providing RANKL signaling, neutrophils can further potentiate the osteoclastogenic effects of the RANKL–RANK axis through the release of multiple pro-inflammatory mediators (97). Activated neutrophils can secrete cytokines such as IL-1β, TNF-α, OSM, and IL-17, which stimulate osteoblasts, fibroblast-like synovial cells, and other immune cells to upregulate RANKL expression while reducing OPG production, thereby further promoting osteoclast differentiation and activation (94, 97). This indirect regulatory pathway enables neutrophils to function not only as a direct source of RANKL, but also as an important amplification node within the osteoclastogenic signaling network in inflammatory bone diseases.Taken together, by combining the direct action of membrane-bound RANKL with the indirect enhancement mediated by pro-inflammatory factors, neutrophils reinforce the RANKL–RANK signaling axis and thereby represent an important mechanism underlying enhanced osteoclastogenesis under inflammatory conditions.

#### 5.1.2 ROS and oxidative stress-driven osteoclastogenesis

Among the multiple mechanisms by which neutrophils promote osteoclastogenesis, ROS production and oxidative stress represent a particularly important pathway. ROS and oxidative stress are now recognized not merely as byproduct of inflammation, but as critical signaling mediators that regulate osteoclast differentiation, activation, and bone-resorptive function (94, 102, 103). Upon activation, neutrophils undergo a pronounced respiratory burst and rapidly generate large amounts of ROS, thereby establishing a strong oxidative stress state within the local microenvironment (104, 105). Neutrophil-derived ROS can act directly on osteoclast precursor cells, promote their differentiation and maturation through redox-sensitive signaling pathways, and enhance the bone-resorptive activity of mature osteoclasts (94, 106, 107).

Mechanistically, ROS activates multiple downstream pathways involved in osteoclastogenesis, among which NFATc1 signaling is of particular importance (108–110). Oxidative stress enhances NFATc1 expression and promotes its nuclear translocation, thereby driving the transcriptional program required for osteoclast differentiation (102, 109). Moreover, RANKL stimulation itself induces ROS generation in osteoclast precursors, creating a positive feedback loop between oxidative signaling and the RANKL–RANK pathway, which further amplifies osteoclastogenic signaling and accelerates osteoclast formation (94, 109, 111).

Under certain pathological conditions, this ROS-dependent regulatory mechanism becomes especially evident (102, 111, 112). For example, during estrogen deficiency, neutrophils are more susceptible to excessive activation, resulting in persistent ROS release and accumulation of oxidative stress in the bone microenvironment, which disrupts oxidative–antioxidative homeostasis (94, 113). This chronic oxidative stress not only promotes osteoclastogenesis and osteoclast activity, but also hinders the reestablishment of bone remodeling balance in favor of osteogenesis, thereby exacerbating bone resorption and contributing to the onset and progression of osteoporosis (94, 114, 115).

#### 5.1.3 NETs-mediated promotion of osteoclastogenesis

NETs represent an important mechanism through which neutrophils may promote osteoclastogenesis. Increasing evidence suggests that NETs can facilitate osteoclast differentiation and enhance osteoclastogenic activity under inflammatory or metabolically dysregulated conditions (94, 95, 100, 116). In particular, NETs may potentiate osteoclast formation and contribute to increased bone resorption, thereby providing an additional link between neutrophil activation and pathological bone loss (94, 100, 116).

Thus, NET formation may represent an important mechanism by which neutrophils promote osteoclastogenesis and disrupt bone remodeling homeostasis. The detailed molecular mechanisms underlying NET-mediated regulation of osteoclastogenesis and bone resorption, as well as its roles in different bone pathological conditions, will be discussed in detail in Section 6.

#### 5.1.4 Chemokines and subset-specific effects on osteoclastogenesis

In addition to direct signaling axes and extracellular effector mechanisms, neutrophils can also fine-tune osteoclastogenesis through chemokine secretion and subset-specific functional heterogeneity (94, 97, 117). As an important source of chemokines in the inflammatory microenvironment, neutrophils provide an additional regulatory layer for osteoclast formation by influencing the recruitment, localization, and differentiation propensity of osteoclast precursors (118–122). Studies have shown that neutrophil-derived C-X-C motif chemokine ligand 1 (CXCL1) can act on CXCR2 expressed by osteoclast precursor cells, directly promoting their differentiation into mature osteoclasts and accelerating bone resorption (94, 118, 123, 124). This effect not only enhances the efficiency of osteoclast formation, but also facilitates the accumulation of osteoclast precursors within the bone microenvironment through the chemotactic axis, thereby creating favorable conditions for sustained bone resorption (94, 119).In addition, the subset composition of bone marrow neutrophils changes substantially with aging or prolonged inflammatory stimulation (125). Recent studies have identified a population of TGF-β1^+^CCR5^+^ neutrophils (TCNs) that is markedly expanded in senescence-associated bone marrow (126, 127). This subset can enhance osteoclast activity and promote bone resorption through local release of TGF-β1, and is therefore considered a potentially important regulatory pathway contributing to bone loss in the context of immunosenescence (126, 128).Collectively, these findings suggest that neutrophils are not functionally uniform regulators of osteoclastogenesis (94, 97). Instead, through chemokine signaling and subset-specific effects, they can exert distinct influences on osteoclast formation under different physiological and pathological conditions (129–131). This multilayered regulation further underscores the pathogenic role of neutrophils in inflammation- and aging-associated disturbances of bone metabolism (98, 126, 128).

### 5.2 Inhibitory effects of neutrophils on osteoclastogenesis

Although the majority of studies support a role for neutrophils in promoting osteoclast responses in inflammation-related bone diseases, limited evidence suggests that neutrophils may suppress osteoclastogenesis under particular developmental or microenvironmental conditions (97, 132, 133). Notably, these observations are derived mainly from basic experimental studies, and the underlying mechanisms appear to be distinct from the pro-osteoclastogenic effects of mature neutrophils in inflammatory contexts (95, 97).

Evidence from bone marrow microenvironment studies suggests that certain neutrophil-associated populations, particularly neutrophil progenitors or incompletely activated neutrophils at early developmental stages, may suppress osteoclastogenesis through the release of inhibitory signals (97). In these settings, neutrophil presence has been linked to reduced osteoclast numbers and decreased expression of osteoclast differentiation markers, indicating that neutrophils are not invariably pro-osteoclastogenic (95, 97, 134, 135). This inhibitory effect appears less likely to depend on direct blockade of the classical RANKL–RANK axis and may instead reflect a homeostatic function of neutrophils in the bone marrow niche, whereby they constrain osteoclast precursor expansion, modulate intercellular interactions, and maintain local immune equilibrium (97, 132, 136, 137).

Current evidence supporting an inhibitory effect of neutrophils on osteoclastogenesis remains limited and is derived mainly from specific experimental models or non-inflammatory settings. Compared with the well-documented pro-osteoclastogenic effects of neutrophils under inflammatory or tissue-injury conditions, these inhibitory observations are more likely to reflect a homeostatic regulatory role for neutrophils during the maintenance phase of bone turnover rather than a dominant function in pathological bone destruction (97).These apparently conflicting findings suggest that neutrophil-mediated regulation of osteoclast responses is highly context-dependent, with its functional outcome determined by the stage of neutrophil differentiation, activation status, and the characteristics of the local microenvironment (58, 95, 97). During active inflammation or tissue repair, neutrophils are more likely to release pro-inflammatory cytokines, enhance ROS production, and promote NET formation, thereby directly or indirectly facilitating osteoclast differentiation and reshaping the local milieu into one that favors osteoclastogenesis and bone resorption (93, 138, 139). By contrast, under non-inflammatory homeostatic conditions, neutrophils may exert a more basal restraining effect on osteoclast responses, thereby preventing excessive bone resorption and contributing to the maintenance of bone remodeling homeostasis (97).

## 6 The role of NETs in bone metabolism

The role of NETs in bone metabolism is best understood as a double-edged sword. Under acute and tightly regulated conditions, NETs may contribute to early tissue repair by providing a transient extracellular scaffold and modulating the local inflammatory microenvironment (95, 140, 141). However, excessive or persistent NET formation is predominantly associated with pathological bone remodeling, promoting osteoclastogenesis, impairing osteogenesis, and disrupting bone homeostasis (100, 142, 143). Thus, the effects of NETs on bone metabolism may depend on their abundance, persistence, and local microenvironmental context. The following sections discuss the effects of NETs on bone resorption, bone formation, and their potential pro-regenerative functions under specific conditions.

### 6.1 The role of NETs in bone metabolism to promote bone resorption

Increasing evidence suggests that NETs function not only as amplifiers of inflammatory responses, but also as direct regulators of osteoclastogenesis and bone resorption, highlighting their important role in bone metabolism (95, 100, 136). Mechanistically, NETs promote the differentiation of osteoclast precursors into mature osteoclasts and enhance the bone-resorptive activity of mature osteoclasts through multiple pathways, which can be broadly divided into RANKL-dependent and RANKL-independent mechanisms (95, 100, 116).

On the one hand, NETs can enhance RANKL-mediated osteoclastogenesis (100). The DNA–protein complexes and granule-derived components released by NETs markedly increase the responsiveness of osteoclast precursors to RANKL signaling, promote activation and nuclear translocation of NFATc1, and upregulate the expression of osteoclast differentiation- and function-related markers such as TRAP and cathepsin K, thereby accelerating osteoclast maturation and promoting bone resorption (100, 144). In addition, NETs can further enhance the bone-resorptive activity of mature osteoclasts, as reflected by expansion of the resorption area and increased formation of resorption pits (100). These findings suggest that NETs not only facilitate osteoclastogenesis, but also directly potentiate osteoclast functional activity (100).

On the other hand, NETs may also promote osteoclast formation through RANKL-independent mechanisms, thereby contributing to bone resorption (95, 116). Studies have shown that histones enriched in NETs, particularly H3 and H4, can function as damage-associated molecular patterns (DAMPs) and activate Toll-like receptor 4 (TLR4) signaling on monocytes, thereby rapidly inducing the formation of osteoclast-like cells even in the absence of exogenous RANKL stimulation (95, 116). This process can occur within a relatively short time *in vitro*, suggesting that NETs possess the capacity to drive osteoclast differentiation by at least partially bypassing the classical RANKL–RANK axis (95, 116).In addition, neutrophil elastase (NE) associated with NETs appears to participate in this process, potentially amplifying osteoclastogenic effects by modulating histone bioactivity or influencing receptor signaling on precursor cells (95, 145). Notably, post-translational modifications of NETs components, such as histone carbamylation, may further enhance their ability to promote osteoclast differentiation, indicating that the biochemical modification state of NET-associated proteins within the inflammatory microenvironment can substantially influence the intensity of NET-mediated bone resorption (95, 145).

Beyond their direct effects on osteoclast precursors, NETs may also indirectly enhance bone resorption by remodeling the immuno-inflammatory landscape of the bone microenvironment (146–148). As DAMPs, NETs can be sensed by a variety of PRRs, leading to activation of local immune responses and amplification of inflammatory signaling (149–151). Persistent NETs production sustains pro-inflammatory cytokine networks, disrupts bone microenvironmental homeostasis, and establishes a permissive inflammatory background for osteoclastogenic responses (95, 136, 152). Moreover, NETs can disturb the homeostatic balance of the RANKL/OPG axis by modulating osteoblast expression of osteoclast-regulatory factors (100). Specifically, NETs stimulation may increase RANKL expression while suppressing OPG production in osteoblasts, thereby elevating the RANKL/OPG ratio, sensitizing osteoclast precursors to RANKL, and ultimately promoting osteoclast differentiation and maturation (100).

Furthermore, NETs-mediated inflammatory amplification promotes the local recruitment of multiple immune cell populations, which in turn release additional pro-osteoclastogenic factors and further sustain NETs formation (145, 153). This creates a positive feedback loop that preserves a pro-inflammatory microenvironment permissive for osteoclastogenesis (153). Together, these findings indicate that NETs indirectly enhance bone resorption by reshaping the bone microenvironment through immuno-inflammatory networks, thereby representing an important mechanism of inflammation-related bone loss (100).

### 6.2 Inhibition of bone formation in NETs

Beyond their role in osteoclastogenesis, NETs can directly suppress bone formation by impairing osteoblast-lineage function through multiple levels of regulation (94, 142, 154). Available evidence indicates that both the magnitude of NETs formation and the activity of NETs-associated signaling pathways are closely correlated with osteogenic differentiation capacity (98, 142, 148). In particular, NCF2-dependent oxidative burst signaling is critically involved in NET generation, and its excessive activation can significantly compromise osteogenic differentiation (155). By contrast, inhibition or knockdown of NCF2 reduces NETs formation and partially rescues NETs-mediated osteogenic defects, indicating that the molecular machinery driving NETosis itself acts as an important upstream regulator of osteogenic potential (155).

Excessive NETosis in the bone marrow microenvironment has been increasingly linked to senescence-associated changes in BMSCs and to impaired osteogenic differentiation (98, 142). Components associated with NETs, including oxidative stress-related signals, inflammatory mediators, and extracellular histones, may function as persistent stressors that drive cell-cycle arrest, mitochondrial dysfunction, and repression of osteogenic transcriptional programs in BMSCs, ultimately reducing bone-forming capacity (98, 142). In line with this, *in vivo* removal of NETs can partially re-establish bone marrow homeostasis, attenuate the senescent phenotype of BMSCs, and restore osteogenic potential, further supporting the pathogenic role of NETs in bone formation impairment (92, 98).

Collectively, excessive or persistent NETs inhibit bone formation at both the level of osteoblast differentiation and the level of stem cell function by impairing osteogenic differentiation and inducing BMSCs senescence (98, 142). Together with their pro-osteoclastogenic effects, these mechanisms shift bone metabolism toward a state of resorption predominance with impaired formation (98, 100, 136). This highlights NETs-targeted intervention, either by limiting NET formation or facilitating the clearance of aberrant NETs, as a potential strategy for restoring bone formation homeostasis.

### 6.3 The role of NETs in bone metabolism to promote bone formation

Although NETs are most commonly linked to enhanced bone resorption in inflammatory contexts, recent studies suggest that they may also contribute to bone formation-related events under specific spatiotemporal conditions, indicating a context-dependent pro-regenerative potential (148, 156, 157). Following their release, NETs form a chromatin-based reticular structure with considerable spatial support and adsorptive capacity, enabling them to serve as a provisional biological scaffold for mineralization-associated processes (140, 158). By concentrating inorganic ions and organic matrix components required for mineral deposition, this structure may help establish a local microenvironment conducive to mineralization and hard tissue formation (158).

NETs may also participate in the formation of a bone-supportive microenvironment by adsorbing and locally enriching a wide range of tissue repair-related bioactive molecules (140, 159). NETs have been reported to capture multiple growth factors and ECM components, thereby creating a relatively stable local biomolecular reservoir that facilitates the recruitment, adhesion, and functional maintenance of osteogenesis-related cells (160). By spatially concentrating these bioactive cues, NETs may influence the spatiotemporal distribution of osteogenic signals and help establish a microenvironment conducive to subsequent bone formation (100, 161, 162).

In this setting, the bone-promoting effect of NETs is not mediated primarily through direct regulation of osteoblastogenesis or osteoblast activity, but rather through their function as a temporary structural scaffold during a short-lived and controllable immune response, together with their capacity to create a microenvironment permissive for bone formation (95, 140, 162).Therefore, the effects of NETs on bone formation should be interpreted in a context-dependent manner, with the duration, magnitude, and local microenvironment of NET formation likely determining whether NETs contribute to tissue repair or pathological bone remodeling.

### 6.4 Contextual determinants of the dual effects of NETs

The biological effects of NETs on bone remodeling appear to depend not only on their abundance, but also on their persistence, clearance, and surrounding microenvironment (163). To integrate these contextual factors, we propose a conceptual framework that distinguishes three functional states of NETs: pro-regenerative scaffolds, a transitional or balanced state, and pro-inflammatory destructive factors (**Table 1**). In the early phase of tissue injury, a transient and controlled increase in NETs formation may contribute to antimicrobial defense, containment of cellular debris, organization of the inflammatory microenvironment, and formation of a temporary extracellular scaffold (140, 164). When NET formation is efficiently resolved, these effects may support the transition from early inflammation toward tissue repair and subsequent bone formation (164). In contrast, when NET accumulation becomes excessive or prolonged, NETs may shift toward a predominantly pro-inflammatory and tissue-destructive state.

**Table 1:** Contextual determinants of the dual effects of neutrophil extracellular traps (NETs) on bone remodeling.

NETs state	NETs level	Duration	Clearance/DNase I activity	ROS state	Functional consequences	Bone outcome	Transition determinants	Representative disease contexts
Pro-regenerative scaffolds	+ → ++	Transient; early peak → decline	Efficient	Transient/controlled	Antimicrobial defense; debris containment; temporary extracellular scaffold; initiation of tissue repair	Favors early tissue repair and subsequent osteogenesis	Balanced neutrophil recruitment; controlled ROS/PAD4 activation; effective NET clearance; pro-resolution signals	Acute fracture repair; early-stage bone injury; early tissue integration around biomaterials
Transition/balanced state	++	Intermediate; gradually resolving	Moderate	Moderate/resolving	NETs-mediated host defense gradually gives way to inflammatory resolution and tissue remodeling	Compatible with normal bone remodeling and repair	Declining neutrophil recruitment; ROS normalization; increasing NET degradation; macrophage-mediated resolution	Intermediate phase of fracture healing; uncomplicated bone regeneration; early implant/tissue healing
Pro-inflammatory destructive factors	++ → +++	Persistent; delayed resolution	Insufficient/impaired	High/sustained	Inflammatory amplification; cellular toxicity; impaired osteogenic signaling; enhanced osteoclastogenic activity	Impaired bone formation and increased bone resorption	Excessive neutrophil activation; sustained ROS/PAD4 signaling; defective NET clearance; chronic inflammatory, infectious, metabolic, or biomaterial cues	Rheumatoid arthritis; periodontitis; chronic osteomyelitis; postmenopausal osteoporosis; aging-associated bone loss; tumor-associated bone disease

Relative NETs abundance, persistence, clearance, local ROS status, and microenvironmental factors may influence whether NETs function as pro-regenerative scaffolds or pro-inflammatory destructive factors. Qualitative descriptors represent relative patterns reported in the literature rather than standardized quantitative thresholds.

The symbols “+” and “++” indicate relatively low and high NETs levels, respectively, whereas “→” indicates a temporal progression or change in NETs levels over the course of the response. These symbols are qualitative descriptors and do not represent quantitative measurements.

Several interacting factors may determine this functional transition. The magnitude and duration of neutrophil recruitment and activation directly influence the local NET burden, while the efficiency of NET degradation and clearance determines how long extracellular NET components persist (165). DNase I-mediated degradation represents an important component of this clearance process, and impaired degradation may favor prolonged NET accumulation (166). ROS availability is another major determinant because NOX2-dependent oxidative bursts can promote NET formation; transient and controlled ROS production may accompany physiological neutrophil activation, whereas sustained oxidative stress may reinforce NETosis and inflammatory signaling (167). PAD4-mediated histone citrullination further contributes to chromatin decondensation and NET formation (168). These processes do not operate independently but are shaped by local and systemic cues, including inflammatory mediators, immune complexes, microbial stimulation, biomaterial surface properties, metabolic status, and neuro-immune regulation (168). Thus, the balance between NET formation and resolution, rather than NET abundance alone, may determine whether NETs function predominantly as a transient regenerative scaffold or as a persistent inflammatory effector that impairs osteogenesis and promotes osteoclast-associated bone resorption.

Importantly, this framework should be interpreted as a conceptual model rather than a quantitatively defined threshold system. Current studies generally report NETs using heterogeneous readouts, including extracellular DNA, MPO-DNA complexes, citrullinated histone H3, or immunofluorescence-based measurements, and these approaches do not provide a standardized measure of the absolute amount or biological activity of NETs. Moreover, the temporal dynamics of NET formation and clearance are incompletely characterized across different bone tissues and disease settings. Although ROS is a recognized upstream driver of NET formation, a specific local ROS threshold that separates beneficial from detrimental NETosis has not been established. Similarly, quantitative rates of DNase I-mediated NET degradation are rarely measured *in vivo* and cannot currently be used to define a universal clearance threshold (169). Therefore, the “+” categories and qualitative descriptors used in **Table 1** are intended to represent relative patterns reported in the literature rather than absolute biological concentrations or fixed temporal thresholds. Future longitudinal studies combining standardized NET quantification, real-time assessment of ROS, measurements of NET degradation and clearance, and bone remodeling outcomes will be necessary to determine whether a quantitative threshold exists for the transition from regenerative to destructive NET activity.

## 7 Key points of evidence for neutrophils in different bone metabolic pathology scenarios

The involvement of neutrophils in bone metabolism varies substantially across different pathological conditions. Rather than exerting a uniform effect, neutrophils undergo disease-specific changes in abundance, activation state, inflammatory phenotype, and effector function, which in turn influence the balance between bone formation and bone resorption. In general, excessive or persistent neutrophil activation is associated with a shift toward bone resorption, whereas alterations in neutrophil homeostasis may contribute to bone remodeling imbalance even in conditions without overt acute inflammation. The following sections summarize the characteristic changes and functional states of neutrophils in representative bone metabolic disorders and their associated effects on bone remodeling. To provide an integrated overview of the available evidence, **Table 2** summarizes representative disease and repair contexts in which neutrophils have been implicated in bone metabolism, together with their functional states, proposed mechanisms, bone outcomes, and levels of evidence. The table also highlights the heterogeneity of neutrophil-mediated effects across pathological and reparative settings.

**Table 2:** Evidence summary of neutrophil-mediated regulation of bone metabolism across disease and repair contexts.

Disease/model	Neutrophil state	Target cell/process	Proposed mechanism	Bone phenotype	Evidence type	Evidence level
Rheumatoid arthritis (95, 100)	Activated, pro-inflammatory neutrophils; increased NET formation	Osteoclasts/osteoclast precursors	NET-associated components can potentiate RANKL-induced osteoclastogenesis; inflammatory neutrophil activity is associated with bone erosion	Increased osteoclastogenesis and bone erosion	Human (100) Animal (95) *In vitro* (95)	Established
Periodontitis (193, 194)	Persistently activated neutrophils; increased NET-associated activity	Periodontal microenvironment/osteoclastogenesis	Persistent neutrophil activation and NET-associated factors may amplify inflammatory signaling and bone resorption	Alveolar bone loss	Human (193) Animal (194)	Established
Chronic osteomyelitis (113, 195)	Persistently activated inflammatory neutrophils	Infected bone microenvironment/osteoclasts	Sustained neutrophil recruitment and inflammatory activity may favor bone resorption and impair formation–resorption coupling	Bone erosion and impaired bone remodeling	Human (113) Animal (195)	Preliminary
Postmenopausal osteoporosis (113)	Dysregulated neutrophil homeostasis; enhanced inflammatory activity and NET formation	Bone remodeling microenvironment/osteoclasts	Estrogen deficiency-associated neutrophil dysregulation and enhanced NETosis may contribute to resorption-dominant remodeling	Increased bone resorption and reduced BMD	Human (113) Animal (113)	Established
Age-related bone loss (98)	Aging-associated/functionally altered neutrophils; increased NETosis	Osteoclasts/bone marrow microenvironment	ROS-associated NETosis and altered neutrophil activity may promote osteoclast activity and bone resorption	Age-related osteoporosis/bone loss	Animal (98) *In vitro* (98)	Established
Tumor-associated bone disease (196, 197)	Tumor-associated, immature or tumor-supportive neutrophils	Tumor–bone microenvironment/osteoclasts	Tumor-derived signals may reprogram neutrophils toward tumor-supportive states, potentially affecting osteoclast-mediated bone remodeling	Tumor-associated bone destruction/osteolysis	Animal (196) *In vitro* (197)	Preliminary
Acute bone injury/early fracture repair (198, 199)	Rapidly recruited, activated neutrophils	Fracture hematoma/MSC recruitment/angiogenesis	Debris clearance, inflammatory signaling, provisional matrix formation, and vascular remodeling may establish a reparative microenvironment	Supports initiation of bone repair	Human (198) Animal (199)	Established
Intermediate-stage fracture healing (66, 199)	Transitioning/repair-associated neutrophils	Angiogenesis/MSCs/inflammatory resolution	Declining inflammatory activity and neutrophil transition toward a repair-associated state may facilitate inflammatory resolution and vascular–osteogenic coupling	Progression toward bone regeneration	Human (66) Animal (199)	Established
Critical-sized calvarial defect (66)	Early recruited, activated neutrophils	BMSCs/bone regeneration	Early neutrophil recruitment may facilitate the initiation of repair and subsequent recruitment of regenerative cells	Reduced new bone formation after neutrophil depletion	Animal (66)	Established
Tibial implant osseointegration (69, 162)	Early recruited neutrophils	BMSCs/osteogenic response/osseointegration	Neutrophil-derived signals may promote BMSC recruitment and early osteogenic responses at the implant interface	Reduced peri-implant bone formation and osseointegration after neutrophil depletion	Animal (69) *In vitro* (162)	Established
IL-8-induced ectopic endochondral ossification (66)	N2-like/repair-associated neutrophils	BMSCs/MSC recruitment	Neutrophil-derived SDF-1α may promote BMSC recruitment through the SDF-1/CXCR4 axis, facilitating bone regeneration	Enhanced ectopic bone formation	Animal (66) *In vitro* (66)	Established
Early biomaterial implantation/tissue integration (200)	Early recruited and activated neutrophils	Biomaterial interface/MSCs/vascular microenvironment	Neutrophil-mediated inflammatory and matrix-remodeling responses may influence MSC recruitment and early tissue integration	Early tissue integration and regeneration	Animal (200) *In vitro* (200)	Preliminary
Diabetic alveolar bone regeneration (91, 201)	Dysfunctional neutrophils; altered ROS and excessive NET formation	Inflammation resolution/angiogenesis/osteogenesis	Dysregulated neutrophil activity and excessive NET formation may delay inflammatory resolution, whereas restoration of neutrophil homeostasis may facilitate vascular–osteogenic repair	Impaired bone regeneration under diabetic conditions	Animal (201) *In vitro* (91)	Established

### 7.1 Bone erosion in rheumatoid arthritis

Rheumatoid arthritis (RA) is a chronic inflammatory joint disease characterized by persistent synovitis and progressive bone erosion (170–172). Neutrophils are among the earliest recruited and most abundant inflammatory cells in RA synovial fluid, and their accumulation is closely associated with disease activity as well as imaging evidence of structural bone damage (95, 100, 173). Compared with non-inflammatory conditions, neutrophil abundance and activation are markedly increased in RA synovial fluid and tissue, reflecting a predominantly activated and pro-inflammatory neutrophil state (95, 100, 173). In addition to increased local accumulation, neutrophils in RA exhibit enhanced effector activity, including increased NET formation and release of inflammatory and tissue-damaging factors (95, 100, 116, 146). The persistence of these activated neutrophils contributes to a sustained inflammatory environment within the synovium and is associated with increased osteoclast activity and progressive bone erosion (95, 100, 116).

From a translational perspective, these findings suggest several potential strategies for the prevention and treatment of RA-associated bone erosion. Targeting NETs formation or enhancing NETs clearance—such as through PAD4 inhibition, attenuation of oxidative stress, or DNase-based therapies—may help restrain pathological osteoclastogenesis (100, 174). Notably, PAD4 may serve as a common therapeutic target in both RA-associated bone erosion and diabetic bone repair, although its pathological roles differ between these settings: it is mainly linked to NET formation and inflammation-driven bone destruction in RA, whereas in diabetic bone repair, PAD4 is associated with neutrophil metabolic dysregulation and impaired osteogenesis (91, 175).Likewise, interventions aimed at NETs-associated modified antigens or proteolytic activity may slow structural bone damage while also mitigating inflammation (145, 176). Moreover, early monitoring of neutrophil accumulation and NET burden in synovial fluid may provide a useful biomarker framework for predicting the risk of erosive progression in RA (149, 177).

### 7.2 Chronic inflammation such as periodontitis and osteomyelitis

In chronic infectious and inflammatory bone diseases, neutrophils play a dual role as both essential antimicrobial effector cells and potential mediators of tissue and bone destruction (178, 179). Their characteristic feature in these conditions is persistent recruitment and activation at sites of infection or inflammation. Although this response is initially protective and necessary for pathogen control, prolonged or dysregulated neutrophil activation can promote excessive inflammation and shift local bone remodeling toward resorption.

In periodontitis, neutrophils constitute the dominant immune cell population within the gingival sulcus and periodontal pockets (178). Both clinical and experimental evidence indicates that local neutrophil accumulation correlates positively with the severity of periodontal tissue destruction (180). Importantly, the pathological effect of neutrophils in periodontitis does not simply reflect their abundance. Rather, persistent activation and an imbalance between antimicrobial activity and inflammatory resolution can result in sustained release of inflammatory and tissue-damaging factors, including NET-associated components such as histones and NE (152, 180). Thus, in the chronic infectious setting of periodontitis, neutrophils and their NETs act not only as antimicrobial effectors, but also as important amplifiers of pathological bone resorption (152).

A similar pattern is observed in osteomyelitis, where neutrophils are extensively recruited to infected bone tissue and remain a prominent component of the inflammatory infiltrate (181). Increased neutrophil accumulation is often associated with elevated osteoclast numbers and more extensive bone erosion (182). In this setting, neutrophils maintain a strong antimicrobial function, but prolonged activation within the infected bone microenvironment may sustain inflammatory signaling and impair the balance between bone formation and resorption (182, 183). Thus, neutrophils in osteomyelitis exhibit a predominantly activated inflammatory phenotype that contributes to a resorption-dominant remodeling state (179, 182).

From a translational perspective, neutrophils in chronic inflammatory bone diseases such as periodontitis and osteomyelitis function not only as essential antimicrobial effectors but also as key synergistic drivers of bone destruction (116, 138). Accordingly, alongside conventional antimicrobial treatment, controlled modulation of excessive neutrophil infiltration or suppression of pathological NET formation may represent a useful strategy for limiting inflammatory bone loss (152, 153). In parallel, therapeutic approaches targeting inflammatory chemokines and NET-associated pathways may offer additional opportunities to restore bone remodeling balance in chronically inflamed environments (152, 184).

### 7.3 Estrogen deficiency-related osteoporosis

In postmenopausal osteoporosis (PMO), neutrophils are increasingly recognized as important immunomodulators involved in the regulation of bone remodeling balance (94, 136). Distinct from overt inflammatory bone destruction, PMO is characterized by low-grade chronic inflammation and immunosenescence, conditions under which neutrophil functional alterations may contribute to the uncoupling of bone formation and bone resorption (94, 113).

Estrogen deficiency is associated with alterations in neutrophil homeostasis and inflammatory activity (94, 113). Consistent with this, an increased neutrophil-to-lymphocyte ratio (NLR) has been reported in postmenopausal women and is inversely associated with bone mineral density, suggesting a relationship between systemic neutrophil-related inflammation and bone loss (94, 96).Mechanistic studies further indicate that estrogen deficiency is accompanied by enhanced NETosis, which is closely associated with increased bone resorption (94, 136). In this setting, excessive NET formation may serve as a key link between immune dysregulation and bone remodeling imbalance (94, 136). Thus, although PMO does not typically present with the extensive neutrophil infiltration observed in infectious or autoimmune bone diseases, neutrophil function appears to be dysregulated under estrogen-deficient conditions.

Functionally, this altered neutrophil state is associated with increased bone resorption and impaired bone remodeling balance (94, 136). Experimental suppression of excessive NET formation or related inflammatory activity can partially alleviate bone loss, supporting the contribution of abnormal neutrophil activation to the resorption-dominant phenotype of estrogen deficiency-related osteoporosis (94, 136). Therefore, neutrophils in PMO are better characterized by functional dysregulation and enhanced inflammatory activity than by overt neutrophil accumulation.

Overall, neutrophil involvement in PMO represents a relatively low-grade but persistent form of immune–bone interaction. Altered neutrophil homeostasis may contribute to the uncoupling of bone formation and bone resorption, ultimately favoring progressive bone loss (94, 98, 136). This disease context highlights that neutrophil-mediated regulation of bone metabolism is not necessarily dependent on massive neutrophil infiltration; changes in neutrophil functional state may also be sufficient to influence long-term bone remodeling.

## 8 Current shortcomings and future directions

Although considerable progress has been achieved in recent years in the study of neutrophil-mediated regulation of bone metabolism, several important gaps in knowledge remain. This review also has several limitations that should be acknowledged. The studies included in this review vary substantially in experimental models, disease contexts, tissue sources, and assessment time points, which limits direct comparison across studies and precludes quantitative synthesis of the available evidence. In addition, some of the proposed mechanisms are supported mainly by *in vitro* or animal studies, whereas direct evidence from human bone tissue remains relatively limited. Therefore, some mechanistic interpretations discussed in this review should be considered as emerging or proposed mechanisms rather than definitively established pathways. Furthermore, this review focuses specifically on the contribution of neutrophils to bone regeneration and metabolism, and therefore does not comprehensively address the broader immune microenvironment that collectively regulates bone remodeling and repair. Neutrophils do not act in isolation, but interact dynamically with macrophages, lymphocytes, osteoclasts, osteoblast-lineage cells, and other immune and stromal populations. Consequently, the effects attributed to neutrophils should be interpreted within the context of the broader immune–bone microenvironment, and future studies should further clarify how neutrophils interact with other immune cell populations to collectively influence bone regeneration. These limitations should be considered when interpreting the current evidence and identifying priorities for future research.

One major limitation of the field is that the functional heterogeneity of neutrophils within the bone microenvironment has not been fully defined. Neutrophils were traditionally viewed as short-lived cells with predominantly acute inflammatory functions; however, growing evidence indicates that they comprise functionally diverse populations that may exert distinct roles during persistent inflammation, tissue repair, and chronic pathological conditions (128). Nevertheless, most current studies continue to treat neutrophils as a single overall population, with insufficient resolution of their developmental trajectory, maturation status, and metabolic characteristics. This population-level perspective restricts a more precise understanding of how specific neutrophil states contribute to bone loss or defective osteogenesis.

Second, the bidirectional role of NETs in bone metabolism remains insufficiently defined. On the one hand, NETs have been shown to amplify inflammatory responses through TLR- and Fc receptor-dependent signaling, promote osteoclastogenesis, and contribute significantly to rheumatoid arthritis and other inflammation-related bone-destructive conditions (149). On the other hand, under certain early injury-repair conditions, moderate NET formation may assist in pathogen clearance, removal of tissue debris, and establishment of a microenvironment favorable for regeneration (140). However, current research has focused mainly on the pathogenic and pro-inflammatory aspects of NETs, and the evidence supporting their pro-regenerative effects remains comparatively limited, with systematic evaluation of their temporal dynamics, persistence, and dose-dependent effects also remaining insufficient (149). Clarifying the conditions under which NET activity shifts from repair-promoting to destruction-promoting will therefore be a key objective for future studies (149).

Importantly, the strength of evidence supporting the proposed bidirectional effects of neutrophils on bone metabolism is not uniform across functional contexts. Evidence for their pro-inflammatory and pro-osteoclastogenic effects is relatively well established across multiple inflammatory and bone-destructive settings, whereas direct evidence for anti-osteoclastogenic effects of neutrophils remains more limited and context-dependent. Similarly, although NETs have been implicated in both tissue repair and bone destruction, evidence supporting their pro-regenerative functions is currently less extensive and is largely derived from specific acute injury or repair settings. These differences in evidence strength should be considered when interpreting the proposed bidirectional roles of neutrophils and highlight the need for further neutrophil-specific and context-defined studies.

Third, the causal link between inflammatory signaling and osteogenesis inhibition has not yet been fully defined. Although substantial crosstalk exists among the NF-κB, MAPK, PI3K–AKT, and Wnt/β-catenin pathways, current evidence is derived largely from *in vitro* studies or single-pathway analyses, with limited *in vivo* characterization of their integrated regulation within the bone microenvironment (185, 186). This issue is especially important in chronic inflammatory settings, where enhanced osteoclastogenesis and suppressed osteogenesis frequently coexist (100). Whether these processes are governed by a shared inflammatory driver or by partially independent regulatory mechanisms remains to be clarified.

In addition, most current animal models are designed to recapitulate a relatively limited number of inflammatory or pathological stimuli, whereas clinical bone disorders usually arise in the context of multiple interacting factors, such as metabolic dysregulation, aging, and altered mechanical loading (187). This gap between experimental models and actual disease states may substantially limit the extrapolation of preclinical findings. Accordingly, the development of more clinically relevant composite models, together with single-cell transcriptomics, spatial omics, and integrative multi-omics approaches, will be essential for a more precise dissection of immune–bone interaction networks within the bone microenvironment (188).

From a therapeutic perspective, although clinical strategies targeting RANKL or inflammatory factors are already available, precise interventions aimed at neutrophils or NETs formation remain in their infancy (189). Future studies should place greater emphasis on selectively restraining pathological inflammation while preserving the physiological functions of inflammatory responses in tissue repair, so as to minimize the risks of over-immunosuppression (190). This will require a shift from indiscriminate blockade toward more precise modulation of the intensity and temporal dynamics of inflammatory signaling (191).

Overall, emerging evidence supports an important role for neutrophils in inflammation-associated bone metabolic imbalance, but the strength of evidence varies across their proposed functions (148). Nevertheless, their functional heterogeneity, temporal dynamics, and signal integration mechanisms remain insufficiently understood. Future progress will depend on the combined application of multi-omics approaches, more clinically relevant model systems, and optimized targeted intervention strategies (91, 148). These efforts are expected to deepen our understanding of immune–bone coupling and ultimately facilitate the development of more precise and individualized therapies for inflammation-related bone disorders (192).

## 9 Summary

As the most abundant innate immune cells in the bone marrow, neutrophils have gradually been redefined from traditional acute inflammatory effectors to key regulatory nodes in the osteoimmune microenvironment (113). Increasing evidence indicates that, rather than serving merely as bystanders during bone metabolism, neutrophils directly or indirectly participate in both osteogenesis and osteoclastogenesis through multilayered signaling networks, thereby exerting profound effects on bone remodeling homeostasis (97, 113).

Neutrophils exert a markedly context-dependent dual effect on osteogenesis. In the early stages of fracture repair or acute injury, appropriately activated neutrophils facilitate osteogenesis initiation by clearing necrotic tissue, promoting angiogenesis, guiding MSCs migration, and remodeling the immune microenvironment (58, 140). By contrast, in chronic inflammation, radiation injury, estrogen deficiency, or aging, neutrophil-derived inflammatory mediators, ROS, and proteases inhibit osteoblast differentiation and function, interfere with osteogenic pathways such as Wnt/β-catenin, and thereby weaken bone formation (94).

In the regulation of osteoclastogenesis, neutrophils are generally regarded as important amplifiers of inflammation-associated bone loss (116). Through expression or induction of RANKL, release of inflammatory mediators such as TNF-α and IL-1β, production of ROS, and formation of NETs, neutrophils reinforce the RANKL–RANK–NFATc1 signaling axis and facilitate osteoclast differentiation via pathways including TLR, Syk, and PI3K–AKT, thereby enhancing bone resorption (94, 95). Notably, persistent NETs can further promote osteoclastogenesis through TLR4/TLR9-mediated mechanisms and by disturbing the RANKL/OPG balance of osteoblast-lineage cells, making them a key pathogenic factor in inflammatory bone destruction and osteoporosis (100). Although neutrophils may exert inhibitory effects on osteoclastogenesis in certain homeostatic contexts, the prevailing evidence suggests that their dominant role in inflammatory conditions is to promote pathological bone resorption (97, 116).

Neutrophils display pronounced temporal specificity, functional plasticity, and microenvironmental dependence in bone metabolism (51, 97). They should not be viewed merely as pro-inflammatory or disease-promoting cells, but rather as a dynamic functional spectrum that undergoes continuous changes across different pathological stages (58). Further elucidation of neutrophil subset characteristics, the regulatory mechanisms of NETs formation, and their crosstalk with osteoimmune signaling pathways will be crucial for revealing the core mechanisms of inflammation-related bone loss (138).

Future studies should focus on developing therapeutic strategies that precisely modulate the magnitude and timing of inflammatory responses, especially through targeting neutrophil functional reprogramming or NETs formation (138). Such approaches may help inhibit pathological bone resorption while preserving the physiological functions of inflammation in tissue repair, thereby offering new theoretical insights and potential therapeutic avenues for osteoporosis and inflammation-related bone destruction (94, 116).
